# The Swedish out-of-home care children cohort (SweOHC) – evaluation of dental health and dental care

**DOI:** 10.1186/s12903-025-06389-1

**Published:** 2025-08-12

**Authors:** Tita Kirkinen, Aron Naimi-Akbar, Andreas Cederlund, Sofia Tranaeus, Gunilla Klingberg

**Affiliations:** 1 Department of Pediatric Dentistry, Faculty of Odontology, Malmö University, Malmö, Sweden; 2 The Clinic of Pediatric Dentistry, Region Värmland, Malmö, Sweden; 3 Health Technology Assessment– Odontology (HTA-O), Faculty of Odontology, Malmö University, Malmö, Sweden; 4 Eastman Dental Institute, Stockholm County Council, Stockholm, Sweden; 5 Swedish Agency for Health Technology Assessment and Assessment of Social Services (SBU), Stockholm, Sweden

**Keywords:** Out-of-home care (OHC) Oral health Dental care needs Registry-based cohort study

## Abstract

**Objectives:**

Children in out-of-home care (OHC) are at greater risk of ill health than other children in the community. The aim of this registry-based cohort study was to compare the oral health and dental care needs of children in OHC with those of other children in Sweden, by merging data from different Swedish registries. A further aim was to analyse whether children in OHC received more dental examinations after 2017, following implementation of a law requiring mandatory health evaluations prior to placement.

**Methods:**

We identified an exposed cohort of Swedish children and young people, 0–19 years old, who had been placed in OHC 2010–2018 (*N* = 50,878), and an unexposed cohort, five times larger, matched for age, sex and county of residence (*N* = 254,380). During the study period, children in OHC received relatively fewer regular, scheduled dental examinations (4.21 vs. 4.88; *p* < 0.0001). More children entering OHC in 2018 received dental examinations (81.7%) compared with 2016 (76.6%) (*p* < 0.0001), but this was still lower than the proportion of controls. Moreover, during the study period, dental caries affected more teeth in children in OHC than in the controls (dft 6-year-olds 1.56 vs. 0.74; *p* < 0.0001, and DFT 12-year-olds 1.18 vs. 0.65; *p* < 0.0001), and they had more extractions and more emergency dental appointments than children who had never been in OHC.

**Conclusion:**

Not only do children in OHC have poorer oral health than other children, they also receive less support from the dental health services. It seems that society has failed in its mission to ensure that children in OHC are not disadvantaged with respect to health and access to comprehensive healthcare. Thus, there is an urgent need for reappraisal of guidelines, legislation, and organizational models for providing dental care to children and adolescents in OHC.

## Introduction

In Sweden, the state has overriding responsibility for the well-being of children in out-of-home care (OHC). However, the living conditions of this vulnerable group of children fall far short of that of other children. At some time during childhood, about 5% of all children in Sweden will be placed in OHC [1]. This is an intervention used by child welfare services, when children are deemed to be at risk of compromised health, or disturbed development, due to their home environment, or their own behavior [1]. Placement may take the form of foster care (including municipal foster homes, emergency placement foster homes, kinship/family network foster homes and also private, consultant-supported foster homes) or institutional settings (municipal group homes, private group homes, or state-run residential care facilities) [2]. Many studies show that children in OHC have more health problems and a greater need for healthcare than their peers [3–11]. The same seems to apply to oral health. Reports from the National Board of Health and Welfare show that children in OHC have poorer oral health and need more extensive treatment for caries but have less frequent dental examinations [12].

The Swedish healthcare system is intended to be socially responsible and equally accessible and is mainly tax funded. According to Swedish law, all citizens have the right to appropriate treatment and healthcare, with priority to those in greatest need [13]. All children and adolescents are entitled to comprehensive health and medical care, free of charge, up to 18 years of age and free dental care, including specialist treatment, up to and including 19 years of age. Between 2019 and 2024, free dental care was extended to include young adults up to the age of 23 years. Dental care is available from either the Public Dental Service or private practitioners. Although children in OHC in Sweden are included in the general healthcare system and health and dental care are free, several studies have shown that these children are not well served by the system [3, 11, 14]. Similar findings are reported in other countries [6, 15] and a systematic review concluded that special organizational solutions are necessary to ensure that children in OHC receive the health and dental care they need and are entitled to [4].

On 1 January 2013, the Social Services Act mandated that Swedish local councils ensure children placed in out-of-home care receive the healthcare they require and are entitled to, with ongoing monitoring of their health status during placement. Since 2017, councils have been required to provide comprehensive health assessments - including somatic (physical), dental, and mental evaluations—for children and young adults up to the age of 20 upon entering out-of-home care [16]. On 1 January 2020, the United Nations Convention on the Rights of the Child (CRC) was adopted as Swedish law, establishing that equal access to healthcare is a fundamental right for all children.

Many previous studies of dental health in children in OHC are based on local, small samples. As there are many high-quality population-based registries in Sweden, registry-based research can be undertaken by combining information from different registries [1]. This is possible thanks to a 12-digit personal identity number (PIN) that is unique for every person registered in the Swedish Population Register. The PIN is provided by the Swedish Tax Agency and consists of numbers which specify the date of birth and sex [17]. The PIN system allows researchers to link data from different registries to a specific individual. There is also a dental health registry including children (The Swedish Quality Registry for caries and periodontal disease (SKaPa) which enables studies on dental health and dental care [18]. The validity of SKaPa has been shown to be high with respect to dft/DFT (number of **d**ecayed, or **f**illed primary **t**eeth/number of **D**ecayed, or **F**illed permanent **T**eeth) and dental treatment [19]. However, data for teeth extracted or missing due to caries, which is included in deft/DMFT (number of **d**ecayed, **e**xtracted (due to caries), or **f**illed primary **t**eeth/number of **D**ecayed, **M**issing (due to caries), or **F**illed permanent **T**eeth), has been reported to be less accurate [20]. Thus, data on caries and filled teeth, but not on extracted or missing teeth, can be used for registry-based research in children [20]. The overall quality of the registries maintained by The National Board of Health and Welfare and Statistics Sweden is regarded as high [1, 21].

There are obvious risks that children in OHC do not receive dental care on the same premises as others and this can impact negatively on their oral health. Thus, in this registry-based cohort study, the aim was to investigate oral health and dental care needs among children in OHC by linking data from different registries.

The specific aims were to address the following questions:

1. Are there differences between children in OHC and other children regarding the dental care they receive (dental examinations, emergency appointments, extractions) and their oral health (dft, DFT, fractures of teeth or jaws)?
2. Has implementation of the new law in 2017 resulted in any differences in frequency of dental examinations in close relation to placement?

### Hypothesis

For many of the children placed in OHC, dental services are not readily accessible. They have poorer dental health and receive less regular dental care than other children.

## Methods

### Study design

This study is based on the Swedish Out of Home Care Children cohort (SweOHC). This registry-based cohort study merged data from different registries provided by The Swedish Quality Registry for caries and periodontal disease (SKaPa) and the registries maintained by The National Board of Health and Welfare and Statistics Sweden. In this study exposed group refers to children placed in out-of-home care (OHC), and unexposed group refers to controls.

### Data sources

Data were sourced from the following registries:

1. **The National Board of Health and Welfare’s registries**:
  - ***National Patient Register***– information about diseases (ICD-10) which can be linked to an increased risk of deteriorating dental health.
  - ***National Register of Measures for Children and Young persons***– to identify the exposed cohort (OHC placement), and information about placements.
2. **Statistics Sweden**:
  - ***The Total Population Register***– to identify the unexposed cohort, and data on deaths and emigration for all research subjects.
  - ***The Longitudinal integrated database for health insurance and labour market studies (LISA)*–** data on socioeconomic variables among the research subjects and their parents.
  - ***The Multi-Generation Register***– to identify the parents of the research subjects.
3. **Swedish Quality Registry for caries and periodontal disease (SKaPa)** – for information about dental health and dental attendance.

### Workflow

**Fig. 1:**
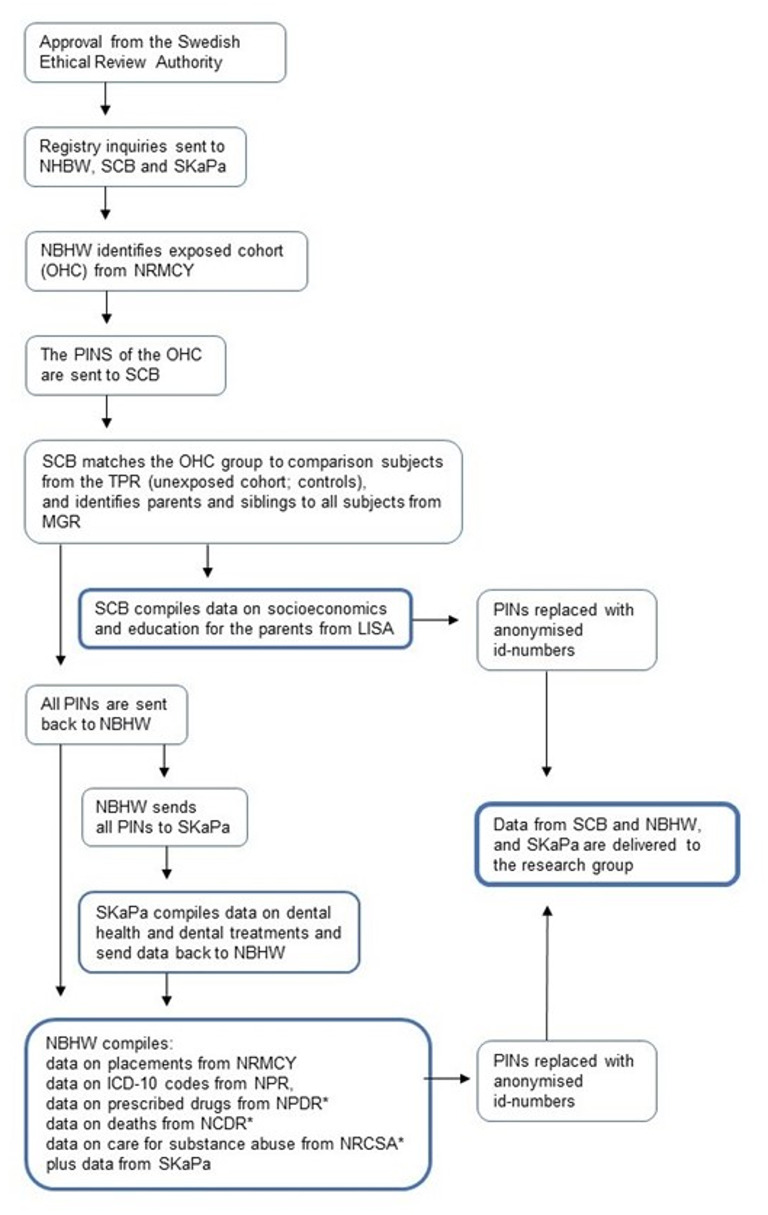
Flowchart of registry inquiry and recruitment of study population. NBHW = National Board of Health and Welfare, SCB = Statistics Sweden, SKaPa = Swedish Quality Registry for caries and periodontal disease, PIN = Personal Identification Number, OHC = children in Out-of-Home Care, NRMCY = National Register of Measures for Children and Young persons, TPR = Total Population Register, MGR = Multi-Generation Register, LISA = Longitudinal integrated database for health insurance and labour market studies, NPR = National Patient Register, NPDR = National Prescribed Drug Register; NCDR = National Cause of Death Register, NRCSA = National Register for Care of Substance Abuse. *=data from registry not used in this paper

The exposed cohort (OHC) was identified by the National Board of Health and Welfare via the ***National Register of Measures for Children and Young persons***. All individuals, aged 0–19 years, who entered OHC at any time during the years 2010–2018 were identified. The National Board of Health and Welfare then sent information about their PINs to Statistics Sweden, whereby the unexposed cohort was constructed from the ***Total Population Register*** by randomly drawing unexposed comparators, in a ratio of 1:5 for each exposed research subject, matched by sex, age and county of residence. To be eligible, the comparators had to be alive and registered in Sweden at the time of inclusion in the study. Statistics Sweden also delivered data on deaths and emigration for the research subjects and identified the parents of both the exposed and the unexposed cohort from the ***Multi-Generation Registry*** and further, produced data on the parents’ socio-economic status from *The****Longitudinal integrated database for health insurance and labour market studies (LISA)***. Statistics Sweden then sent PINs for both the unexposed and the exposed cohort to the National Board of Health and Welfare who retrieved data from SKaPa. The information from SKaPa comprised dental health and dental attendance records for all research subjects, from 2010 to 2020. SKaPa data is based on codes registered in the dental records, with unique codes for different diagnoses, examinations and treatments. All data delivered to the researchers were de-identified, without PINS, but with serial numbers, which enabled merging of the datafiles (Fig. 1).

### Participants

The subjects comprise an exposed cohort of children placed in OHC and an age- and gender-matched unexposed cohort, representing the total population. The exposed cohort comprises all children and adolescents, 0–19 years old, who were placed in OHC in Sweden sometime during the period 2010–2018. The unexposed cohort comprises children and adolescents who are not part of the exposed cohort, matched, at a ratio of 1:5, to the exposed research subjects, according to gender, age and county of residence. Children with a verified status as asylum-seeking unaccompanied minors were excluded in both groups. Children in the exposed group were stratified into three different groups, depending on how long they had been in OHC (total time in OHC):

- 1 = total time in OHC less than one year
- 2 = total time in OHC 1–3 years
- 3 = total time in OHC more than 3 years
- 0 = never placed in OHC (controls)

The stratification of these cutoff points was decided within the research group, based on the experience that both placement duration and the number of placements have an impact on health. This is also supported by reports of an association between shorter placement times and poorer oral health [12]. In order to allow comparisons with official Swedish dental health statistics, five specific age groups, 3-, 6-, 12-, 17-, and 19-years, were used for analyses of dental health.

This is the first study to describe our cohort, the Swedish Out of Home Care Children cohort (SweOHC). Analyses of data from the cohort will continue and be presented subsequently.

### Statistics

Categorical outcome variables are presented as numbers and proportions as percentages for the different exposure groups. Continuous and discrete variables are presented as mean values, with 95% confidence intervals and standard deviations. The two-sample t-test was applied to analyse differences between exposed (children in OHC) and unexposed controls with reference to the number of examinations and emergency appointments, dft/DFT and extractions. In addition, linear regression analyses were used to compare subgroups of OHC with controls, with reference to dental examinations, emergency appointments and dft/DFT. The Chi square test was applied to compare the number of ICD-10 diagnoses and examinations at time of placement in OHC for individuals in OHC and the controls. To adjust for potential confounding factors when analysing the association between OHC and dft/DFT, a multiple linear regression analysis was applied, the model included the different exposure levels of OHC as dichotomous indicator variables and the controls was left out as reference category, parental educational level dichotomous indicator variables with the lowest level as reference, country of birth was included as dichotomous indicator variables with Sweden as reference. The model also included diabetes, mood disorders, mental and behavioral disorders, conduct disorders and hyperkinetic disorders, all as dichotomous variables (yes/no). STATA SE 15 software (Stata Corporation LLC, College Station, U.S.A) was used for all statistical analyses. P-values less than 0.05 was considered statistically significant.

## Results

The total population comprised 409,638 individuals, of whom 68,273 had been placed in OHC at some point during the period 2010–2018. These figures included 8,925 immigrants with the status of unaccompanied minors: 88% were boys, most born in 1999 and 2000. As unaccompanied minors, this group was considered not to have had access to dental health care on the same premises as others, and so they and their controls were excluded: 8,925 exposed children and 44,625 controls (a total of 53,550 individuals). Moreover, 10 controls were excluded because they had the status of unaccompanied minors. Further, only individuals with their first placement in 2010 or later were included why the final study population comprised 305,258 children (50,878 OHC and 254,380 controls) born between 1996 and 2016 (Table 1). In the SKaPa registry, registrations of dental procedures were found for 271,609 individuals (45,413 OHC and 226,196 controls). The characteristics of the study population are shown in Table 2. The mean number of placements in the OHC group was 2,85 with considerable variation in numbers among children in the OHC groups (Table 3).

**Table 1:** Study population by year of birth. Control group and children in out-of-home care (OHC), stratified according to duration of placement: less than 1 year, 1-to-3 years, more than 3 years, and all children placed in OHC

Year of birth	Duration of placement in OHC				
	Controls	Total OHC	OHC < 1yr	OHC 1–3 yrs	OHC > 3yrs
1996	20,720	4,144	1,310	1,117	1,717
1997	22,145	4,429	1,444	1,181	1,804
1998	24,345	4,869	1,493	1,371	2,005
1999	24,653	4,931	1,371	1,345	2,215
2000	22,129	4,427	1,270	1,256	1,901
2001	17,814	3,563	1,035	947	1,581
2002	14,244	2,849	897	744	1,208
2003	11,625	2,325	830	540	955
2004	10,010	2,002	813	397	792
2005	8,180	1,636	618	296	722
2006	8,050	1,610	683	262	665
2007	7,860	1,572	675	238	659
2008	8,085	1,617	689	230	698
2009	8,165	1,633	677	232	724
2010	9,335	1,867	817	224	826
2011	8,090	1,618	700	182	736
2012	8,035	1,607	671	204	732
2013	6,670	1,334	575	168	591
2014	5,670	1,134	489	142	503
2015	4,860	972	412	112	448
2016	3,695	739	300	86	353
Total	254,380	50,878	17,769	11,274	21,835

**Table 2:** Baseline characteristics of the study population. Control group and children placed in out-of-home care (OHC), less than 1 year; 1-to-3 years, more than 3 years, and all children placed in OHC. First placement year 2010 or after

	Controls	Children in out of home care			
	*N* = 254,380 (%)	Total OHC *N* = 50,878 (%)	OHC < 1yr *N* = 17,769 (%)	OHC 1-3yrs *N* = 11,274 (%)	OHC > 3yrs *N* = 21,835 (%)
**In SKaPa**					
	226,196 (88.92)	45,413 (89.26)	15,881 (89.37)	9,794 (86.87)	19,738 (90.40)
**Sex**					
Female	109,294 (42.96)	21,859 (42.96)	8,662 (48.75)	4,396 (38.99)	8,801 (40.31)
Male	145,086 (57.04)	29,019 (57.04)	9,107 (51.25)	6,878 (61.01)	13,034 (59.69)
**Parents’ educational level**	Mothers Fathers				
Primary/lower	71,287 (28.02)	22,088 (43.41)	8,569 (48.22)	3,917 (34.74)	9,602 (43.97)
secondary school	98,239 (38.62)	21,104 (41.47)	8,273 (46.56)	3,789 (33.61)	9,042 (41.40)
Upper	58,204 (22.88)	6,253 (12.29)	2,714 (15.27)	1,152 (10.22)	2,387 (10.93)
secondary school	50,345 (19.79)	4,918 (9.67)	1,964 (11.05)	902 (8.00)	2,052 (9.40)
Post-secondary	115,923 (45.57)	6,274(12.33)	3,112 (17.51)	1,223 (10.84)	1,939 (8.88)
school	88,102 (34.64)	5,315 (10.45)	2,653 (14.93)	1,035 (9.18)	1,627 (7.46)
Data	8,966 (3.52)	16,263 (31.96)	3,374 (18.99)	4,982 (44.19)	7,907 (36.21)
unavailable	17,694 (6.96)	19,541 (38.41)	4,879 (27.46)	5,548(49.21)	9,114 (41.74)
**Country of birth**					
Sweden	227,293 (89.35)	30,355 (59.66)	11,964 (67.33)	5,412 (48.00)	12,979 (59.44)
Other Nordic countries	1,492 (0.59)	269 (0.53)	114 (0.64)	52 (0.46)	103 (0.47)
Other European countries	6,631 (2.61)	1,400 (2.75)	612 (3.45)	287 (2.55)	501 (2.29)
Non-European countries	18,939 (7.43)	18,817 (36.99)	5,066 (28.51)	5,517 (48.94)	8,234 (37.72)
Data unavailable	25 (0.01)	37 (0.07)	13 (0.07)	6 (0.05)	18 (0.08)

**Table 3:** Study population stratified by frequency of placements. Children placed in out-of-home care (OHC) for less than 1 year, 1-to-3 years, more than 3 years, and all children placed in OHC

	Mean	SD	Min -Max	1 placement *N* (%)	2–4 placements *N* (%)	> 5 placements *N* (%)
OHC < 1yr						
*N* = 17,769	1.67	1.06	1–12	11,226 (59.93)	6,053 (25.98)	490 (5.59)
OHC 1–3 yrs						
*N* = 11,274	3.21	2.32	1–29	2,660 (14.20)	6,199 (26.51)	2,415 (27.56)
OHC > 3 yrs						
*N* = 21,835	3.65	2.94	1–36	4,845 (25.87)	11,132 (47.61)	5,858 (66.85)
Total OHC *N* = 50,878	2.85	2.47	1–36	18,731 (36.82)	23,384 (45.96)	8,763 (17.22)

### Medical and psychiatric diagnoses

ICD-10 diagnoses from the medical system, retrieved from the National Patient Register, showed similar frequencies of diabetes in both OHC group and controls. However, children in OHC were diagnosed more frequently with mental and behavioural disorders (41,47% vs. 18.48%; *p* < 0.0001). Similarly, both boys and girls in the OHC group had higher frequencies of kinetic and conduct disorders, as well as mood disorders (Table 4).

**Table 4:** Study population stratified according to ICD-10 diagnoses retrieved from the National patient register. Control group and children placed in out-of-home care (OHC) for less than 1 year, 1-to-3 years, more than 3 years, and all children placed in OHC. Chi-square tests for comparisons between controls and total OHC group

Diagnosis ICD-10	Controls		Children in out-of-home care		*p*-value	
	*N* = 25,4380 (%)	Total OHC *N* = 50,878 (%)	OHC < 1yr *N* = 18,423 (%)	OHC 1-3yrs *N* = 12,142 (%)	OHC > 3yrs *N* = 28,783 (%)	
**Fracture of tooth (S02.5)**						
Female	108 (0.10)	22 (0.10)				0.399
Male	254 (0.18)	84 (0.29)				< 0.0001
Total	362 (0.14)	106 (0.21)	38 (0.21)	12 (0.11)	56 (0.26)	< 0.0001
**Fracture of malar and maxillary bones (S02.4)**						
Female	28 (0.03)	10 (0.05)				0.077
Male	131 (0.09)	60 (0.21)				< 0.0001
Total	159 (0.06)	70 (0.14)	19 (0.11)	20 (0.18)	31 (0.14)	< 0.0001
**Fracture of mandible (S02.6)**						
Female	54 (0.05)	23 (0.11)				0.001
Male	214 (0.15)	75 (0.26)				< 0.0001
Total	268 (0.11)	98 (0.19)	34 (0.19)	22 (0.20)	42 (0.19)	< 0.0001
**Any diagnosis S02.4-S02.6**						
Female	181 (0.17)	51 (0.23)				0.005
Male	569 (0.39)	206 (0.71)				< 0.0001
Total	750 (0.29)	257 (0.51)	88 (0.50)	53 (0.47)	116 (0.53)	< 0.0001
**Diabetes mellitus (E10-E14)**						
Female	792 (0.72)	183 (0.84)				0.092
Male	1,222 (0.84)	187 (0.64)				0.051
Total	2,014 (0.79)	370 (0.73)	112 (0.63)	98 (0.87)	160 (0.73)	0.693
**Hyperkinetic disorders (F90)**						
Female	5,231 (4.79)	4,134 (18.91)				< 0.0001
Male	11,151 (7.69)	5,596 (19.28)				< 0.0001
Total	16,382 (6.44)	9,730 (19.12)	3,018 (16.98)	2,197 (19.49)	4,515 (20.68)	< 0.0001
**Conduct disorders (F91)**						
Female	317 (0.29)	959 (4.39)				< 0.0001
Male	947 (0.65)	1,560 (5.38)				< 0.0001
Total	1,264 (0.50)	2,519 (4.95)	703 (3.96)	637 (5.65)	1,179 (5.40)	< 0.0001
**Mood [affective] disorders (F30-F39)**						
Female	6,102 (5.58)	3,679 (16.83)				< 0.0001
Male	4,535 (3.13)	2,289 (7.89)				< 0.0001
Total	10,637 (4.18)	5,968 (11.73)	1,917 (10.79)	1,599 (14.18)	2,452 (11.23)	< 0.0001
**Mental and behavioural disorders (F00-F99)**						
Female	18,968 (17.36)	9,663 (44.21)				< 0.0001
Male	28,036 (19.32)	11,437 (39.41)				< 0.0001
Total	47,004 (18.48)	21,100 (41.47)	6,758 (38.03)	4,917 (43.61)	9,425 (43.16)	< 0.0001

### Dental examinations and emergency appointments

Throughout the study period, children who had been placed in OHC underwent fewer dental examinations than the controls (Table 5). The opposite applied to emergency attendance: 19.24% of the OHC subjects have had at least one emergency appointment, compared with 14.71% of the controls (*p* < 0.0001) (Table 6).

**Table 5:** Mean number, standard deviation (SD) and 95% confidence interval (CI) of dental examinations in OHC and control groups during the study period. Data shown for controls, children placed in out-of-home care (OHC) for less than 1 year, 1-to-3 years, more than 3 years, and all children placed in OHC. Linear regression analyses comparing different OHC groups with controls, Two-sample t-tests for comparisons between controls and total OHC group

Group	*N*	Mean	SD	95% CI	*p*-value
Controls	226,196	4.88	2.04	4.88–4.89	
OHC < 1yr	15,881	4.44	2.20	4.41–4.48	< 0.0001
OHC 1-3yrs	9,794	3.96	2.16	3.96–4.01	< 0.0001
OHC > 3yrs	19,738	4.14	2.16	4.11–4.17	< 0.0001
Total OHC	45,413	4.21	2.18	4.19–4.23	< 0.0001

**Table 6:** Mean number, standard deviation (SD) and 95% confidence interval (CI) of emergency dental appointments for children placed in out-of-home care (OHC) and control group. Children placed in out- of-home care (OHC) for less than 1 year, 1-to-3 years, more than 3 years, and all children placed in OHC. Linear regression analyses comparing different OHC groups with controls, Two-sample t-tests for comparisons between controls and total OHC group

Group	*N*	Mean	SD	95% CI	*p*-value
Controls	226,196	0.198	0.563	0.196–0.200	
OHC < 1yr	15,881	0.270	0.661	0.259–0.280	< 0.0001
OHC 1-3yrs	9,794	0.307	0.724	0.292–0.321	< 0.0001
OHC > 3yrs	19,738	0.263	0.658	0.253–0.272	< 0.0001
Total OHC group	45,413	0.275	0.674	0.268–0.281	< 0.0001

The proportion of children in the OHC group undergoing dental examinations in close proximity to their placement in OHC (during the year of placement or the year after), was lower than the proportion of controls examined during the same period. When the new law on mandatory health examinations was implemented in 2017, more children in OHC underwent dental examinations (an increase from 76.64% in 2016 to 81.68% in 2018; *p* < 0.0001). During this period, there was a corresponding increase in dental examinations in the controls. Despite this, for all years there was a lower frequency of examinations in OHC compared to the controls (Table 7).

**Table 7:** Numbers and frequencies of children in out-of-home care (OHC) and their controls undergoing dental examinations during the year of placement or the following year. Placements in 2016, 2017 and 2018 and for control group and children placed in OHC for less than 1 year, 1-to-3 years, more than 3 years, and all children placed in OHC. Chi-square tests comparing controls and total OHC group

Year of placement	Examination Yes/No	Controls *N* (%)	Total OHC *N* (%)	OHC < 1yr *N* (%)	OHC 1-3yrs *N* (%)	OHC > 3yrs *N* (%)	*p*- value
**2016**	Yes	26,503 (80.20)	4,956 (76.64)	1,471 (77.34)	860 (78.11)	2,625 (75.78)	< 0.0001
	No	6,542 (19.80)	1,511 (23.36)	431 (22.66)	241 (21.89)	839 (24.22)	
**2017**	Yes	18,847 (84.41)	3,388 (80.61)	781 (79.45)	1,330 (80.17)	1,277 (81.81)	< 0.0001
	No	3,482 (15.59)	815 (19.39)	202 (20.55)	329 (19.83)	284 (18.19)	
**2018**	Yes	24,200 (85.27)	4,552 (81.68)	2,195 (81.66)	1,223 (79.26)	1,134 (84.50)	< 0.0001
	No	4,180 (14.73)	1,021 (18.32)	493 (18.34)	320 (20.74)	208 (15.50)	

### Oral health

Dental caries was studied separately for all children, at the ages of 3-, 6-, 12-, 17-, and 19-years. In all age groups, children in OHC had statistically significantly higher mean dft/DFT values than those who have never been in OHC (Table 8). The findings were similar for the proportion of caries-free children (dft or DFT = 0). For 3-year-olds, 91% of the children in OHC were caries-free, compared with 96% of the controls (*p* < 0.0001). For the 6- year-olds, the corresponding figures were 62% caries free in OHC and 79% in controls (*p* < 0.0001), for 12-year-olds, 53% and 67% respectively, (*p* < 0.000), for 17-year-olds, 25% and 43% respectively, were caries-free (*p* < 0.0001) and for 19-year-olds, 19% of the OHC group were caries free, compared with 37% of controls (*p* < 0.0001).

**Table 8:** Mean number, standard deviation (SD) and 95% confidence interval (CI) for dental caries expressed as Dft or DFT (decayed and filled teeth in primary and permanent dentition, respectively) for children aged 3-, 6-, 12- and 19 years of age. Comparisons within age groups between control group and children placed in out of home care (OHC) less than 1 year, 1-to-3 years, more than 3 years, and all children placed in OHC). Linear regression analyses comparing different OHC groups with controls, Two-sample t-tests for comparisons between controls and total OHC groups

			dft			
Group		*N*	Mean	SD	95% CI	*p*-value
*3 yrs old*						
	Controls	26,608	0.132	0.793	0.122–0.141	
	OHC <1yr	2,035	0.406	1.501	0.341–0.472	< 0.0001
	OHC 1-3yrs	504	0.274	1.247	0.165–0.383	< 0.0001
	OHC > 3yrs	2,322	0.282	1.209	0.232–0.331	< 0.0001
	Total OHC	4,861	0.333	1.344	0.295–0.371	< 0.0001
*6 yrs old*						
	Controls	42,016	0.738	1.880	0.720–0.756	
	OHC <1yr	3,551	1.747	2.760	1.656–1.837	< 0.0001
	OHC 1-3yrs	967	1.570	2.607	1.405–1.734	< 0.0001
	OHC > 3yrs	3,831	1.378	2.521	1.298–1.458	< 0.0001
	Total OHC	8,349	1.557	2.640	1.500–1.614	< 0.0001
			**DFT**			
**Group**		**N**	**mean**	**SD**	**95% CI**	**p-value**
*12 yrs old*						
	Controls	43,668	0.652	1.226	0.641–0.664	
	OHC <1yr	3,390	1.172	1.732	1.114–1.231	< 0.0001
	OHC 1-3yrs	1,291	1.353	1.892	1.250–1.457	< 0.0001
	OHC > 3yrs	4,090	1.136	1.719	1.083–1.188	< 0.0001
	Total OHC	8,771	1.182	1.752	1.145–1.219	< 0.0001
*17 yrs old*						
	Controls	104,69	1.792	2.525	1.777–1.807	
	OHC <1yr	5,945	3.231	3.497	3.142–3.320	< 0.0001
	OHC 1-3yrs	5,299	3.435	3.547	3.340–3.531	< 0.0001
	OHC > 3yrs	9,179	3.305	3.484	3.233–3.376	< 0.0001
	Total OHC	20,423	3.317	3.505	3.296–3.365	< 0.0001
*19 yrs old*						
	Controls	95,109	2.216	2.919	2.198–2.235	
	OHC <1yr	5,231	4.087	4.045	3.977–4.196	< 0.0001
	OHC 1-3yrs	4,687	4.259	4.071	4.143–4.376	< 0.0001
	OHC > 3yrs	7,710	4.066	3.956	3.978–4.154	< 0.0001
	Total OHC	17,628	4.124	4.014	4.064–4.183	< 0.0001

A model wherein parental education, country of birth, psychiatric diagnoses, diabetes and OHC group were applied as explanatory variables on dental caries revealed that in all age groups, an important variable for dental caries was OHC. The adjusted and unadjusted results for 6- and 17-year-olds are presented in Table 9.

**Table 9:** Associations between OHC (placement in out-of-home care) and dft/dft at 6 years and 17 years of age were analyzed by multiple linear regression. Both unadjusted and adjusted regression coefficients are presented with 95% CIs and p- values. The model was adjusted for country of birth, mother’s education, father’s education, diabetes, mood disorders, mental and behavioural disorders, conduct disorders and hyperkinetic disorders

	dft 6 year olds							
	Unadjusted				Adjusted			
	Regression coeff.	95% CI		*p*-value	Regression coeff.	95% CI		*p*-value
Controls	ref.				ref.			
OHC < 1yr	1.009	0.939	1.078	< 0.0001	0.119	0.047	0.191	0.001
OHC 1-3yrs	0.832	0.703	0.961	< 0.0001	-0.107	-0.234	0.019	0.096
OHC > 3yrs	0.64	0.573	0.707	< 0.0001	-0.277	-0.352	-0.203	< 0.0001
	**DFT 17 year olds**							
	**Unadjusted**				**Adjusted**			
	**Regression coeff.**	**95% CI**		**p-value**	**Regression coeff.**	**95% CI**		**p-value**
Controls	ref.				ref.			
OHC < 1yr	1.439	1.368	1.510	< 0.0001	0.685	0.614	0.757	< 0.0001
OHC 1-3yrs	1.643	1.568	1.718	< 0.0001	0.867	0.788	0.946	< 0.0001
OHC > 3yrs	1.513	1.455	1.570	< 0.0001	0.683	0.618	0.748	< 0.0001

dft = decayed and filled teeth in primary dentition DFT = decayed and filled teeth in permanent dentition

**Fig. 2:**
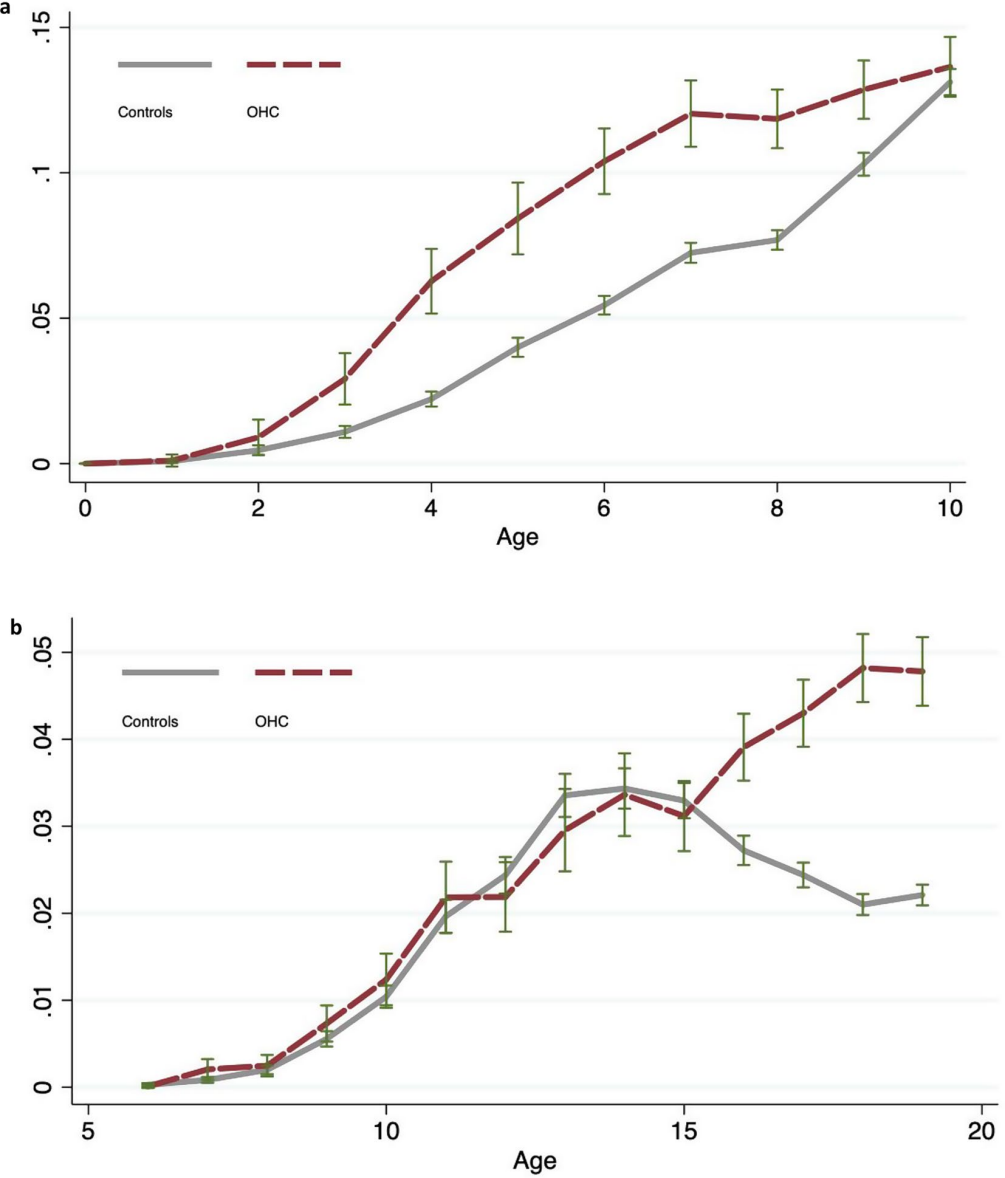
**a and b.** Mean number and 95% confidence intervals of extracted primary teeth **(a)** and permanent teeth **(b)** according to age, in children placed in out-of-home care (OHC) after placement, and unexposed controls

Children in OHC had undergone more extractions than the controls. This applied to both the primary and permanent dentitions (Table 10). As shown in Fig. 2a, the OHC group had more extractions of primary teeth during the entire period of time in which the primary dentition predominates. In contrast, there was an increase in extractions of permanent teeth in the OHC group in their late teens (Fig. 2b).

**Table 10:** Mean number, standard deviation (SD) and 95% confidence interval (CI) of tooth extractions for children placed in out-of-home care (OHC) and control group. Data for primary teeth refer to children born 2008 to 2012; and data for permanent teeth to children born 1996 to 2004. Only data after first placement in OHC. Two-sample t-test

Group	*N*	Mean	SD	95% CI	*p*-value
*Primary teeth*					
Total OHC group	7,881	0.660	1.425	0.629–0.692	< 0.0001
Controls	38,486	0.466	1.106	0.455–0.478	
*Permanent teeth*					
Total OHC group	28,908	0.187	0.648	0.179–0.194	< 0.0001
Controls	145,142	0.119	0.554	0.116–0.121	

Moreover, traumatic injuries to teeth and jaws, diagnosed within medical care, were more common in the OHC group. Overall, children in the OHC group had a mean number of tooth and jaw fractures of 0.51, compared with 0.29 in the controls (*p* < 0.0001). The differences applied to all diagnoses for boys and for the total group. With respect to girls, no differences emerged between those in OHC and those in the control group in terms of the number of tooth fractures (Table 4).

## Discussion

This study shows that children in OHC have more dental caries, more extractions and more dental emergency appointments than children who have never been in OHC. At the same time children in OHC undergo fewer regular, scheduled dental examinations than other children. After the 2017 implementation of a new law on compulsory health assessments at the time of placement, the number of such examinations increased for OHC. However, the number of dental examinations remained lower than for controls.

It is apparent from this study that from many perspectives, children in OHC constitute a vulnerable group. This includes background social factors, for example compared to other children, there are fewer parents with higher levels of education and more children of immigrant background, as well as higher frequencies of psychiatric disorders. There was also a high number of changes of placement in the OHC group. Even among those whose total time in OHC was less than 1 year, there could be up to 12 different placements, which is remarkably high. Altogether, it is highly likely that these factors could affect oral health, as described in several previous studies [22–24]. The present study clearly supports these findings. Not only do children in OHC have more oral health problems, such as caries or trauma, but they also receive less support from dental services. Most obvious is the fact that they receive fewer regular dental examinations.

According to law, children about to be placed in OHC should undergo a dental examination and it is the responsibility of the municipalities and their social services to ensure this [16]. It is acknowledged that children in OHC run a higher risk of health problems. As society assumes responsibilities which usually rest with parents, society must also ensure that children in OHC are not disadvantaged. It is not an easy task, but the results of the present study indicate that society has failed to meet this obligation, a finding which is supported by previous research [4, 6].

The reasons for placing a child in OHC can vary from lack of parental care, unfavourable domestic conditions due to violence or drug abuse, to issues related to the child itself, e.g., behavioural problems, use of drugs or criminal activity [2, 5, 25]. It is understandable that many of the children in OHC do not prioritise their oral health care and that their self-care and dietary habits may be less conducive to oral health. Support from the foster family, or from staff at OHC is of course a key element in health prevention, but these children also need regular dental examinations, treatment planning and possibly treatment. If these needs are not met, oral health will be even more adversely affected.

Fewer dental examinations have been reported previously in OHC children [12], but here we were able to show that even the new law did not ensure that all children in OHC underwent a dental examination, even at the time of placement. One reason for the low number of examinations could be that many children in OHC move between different types of OHC, from OHC to their original family and back to OHC. They also move beyond their municipality or county region. Such numerous moves result in their inability to establish continuity of their relationship with the dental team or dental clinic. Further, the dental recall system does not cater for relocation by patients: it is difficult or even impossible for the system to keep track of the children. For these children there is a need for individualised transfer of dental health information, case history and treatment plans between dental clinics at the locations where the children are placed or live. No such system is currently in place, and it is unclear who should be responsible, social services or the dental care system? At present, no one is responsible and so children in OHC fall through the cracks.

Nonetheless, as social services are involved in the decisions about placement and have access to the address and geographic location of the placement, it is fair to state that they should always send priority letters to both those responsible at the OHC-accommodation and to the dental services when these children move, so that dental care is always ensured. Although dental care is free of charge for all children and young people there is a problem if the child moves to another region for placement in OHC but remains registered in the municipality of the family home. Not only is there is a risk for delay related to a decision as to which region should assume responsibility for providing dental care, but questions also arise about reimbursement. The new region wants to be paid by the home region, which in turn must have a cost estimate that is approved. Often dental care is delayed until this is solved. This is of course unacceptable from the health perspective: these children should simply receive their dental care where they currently live, without bureaucratic obstacles.

This combination of less frequent dental visits, suboptimal self-care and delayed treatment needs will inevitably raise the risk of deterioration in dental health and a need for more advanced dental treatment in both the shorter and longer perspectives. The higher number of emergency appointments for children in OHC disclosed in the present study indicates that lack of regular dental examinations leads to health issues associated with unmet treatment needs. In the present study, this was confirmed by higher numbers of dft/DFT and also in more extractions than in the control children, who received more regular dental examinations. Although the study shows differences between OHC and controls in background factors such as parents’ educational levels, immigrant background and psychiatric health problems, the multiple linear regression analyses showed that OHC and the duration of placement were strong explanatory factors for dental caries.

Taking into account the complex case history, including placement in OHC itself, plus social factors, mental health issues and dental caries status, dentists should assess the risk of oral health problems in children in OHC as generally very high. In accordance with the risk assessment, the treatment plan should include preventive strategies as well as shorter recall intervals. When it is time to contact the patient to schedule check-ups or regular dental examinations, the administrative system should preferably generate an alert to the effect that this a high-risk patient who needs extra attention. Unfortunately, the present study indicates that this routine does not seem to work.

The timing of tooth extractions varied between children in the OHC and control groups. The differences were not analysed in detail but looking at dft/DFT values offers some possible explanations. In the primary dentition dft values were considerably higher in the OHC groups and it is likely that some of the primary teeth were so badly broken down due to caries that they had to be extracted, especially if dental attendance was less frequent. There was greater variation in extractions of permanent teeth, although OHC had more dental caries. In the mixed dentition, up to around the age of 15 years, there were more extractions of permanent teeth among the controls, whereas after 15 years of age there were substantially more extractions in the OHC group. There could be several possible explanations. Compared with the OHC group, controls maybe underwent other types of dental treatment, e.g., more orthodontic treatment as they had lower caries risk, more regular dental care and more stable living conditions. Orthodontic treatment often involves extraction of permanent teeth. Children in OHC are more likely to have had permanent teeth extracted due to caries: this often occurs after the age of 15, by which time orthodontic extractions are less common. Increased numbers of tooth extractions have also been reported in young adults who had been in OHC during childhood [26]. Thus, tooth extractions may serve as an indicator that children in OHC not only have more dental health problems but are also offered different treatment from that of children who have not been placed in OHC. This also raises an issue with respect to the rights of all children to have equal access to health services.

In this study the number of diagnoses related to traumatic injuries was higher in the OHC group. However, as these data were retrieved from the medical health system and not from dental records, it is likely that the information on tooth fractures in particular is underestimated. In most cases, patients incurring traumatic dental injuries will seek dental treatment. Unfortunately, the Swedish dental care system does not provide codes for trauma diagnoses and the information is not retrievable from the SKaPa registry. It was therefore not possible to study this further. Jaw fractures will however, most likely be treated by the medical sector, and this study clearly shows that children in OHC, boys in particular, present with fractures to both the maxilla and mandible more often than other children. The reason for this was not investigated in this study, but it may be hypothesised that the circumstances underlying admission to OHC, e.g., hyperkinetic and conduct disorders could contribute.

Further, it is important to be aware of the strong correlation between lower socioeconomic status and poor dental health in children [27]. The present study did not have data on the children’s dental health before placement in OHC and cannot determine whether the OHC group had higher dft/DFT scores or more emergency visits already prior to placement. However, children in OHC in Sweden have previously been reported to have poorer dental health at the time of their first placement [12]. This may reflect the child’s life situation prior to placement where they are exposed to a combination of socioeconomic and health-related risk factors. This is supported in the present study which clearly demonstrates that the OHC group faces more socioeconomic challenges compared to the control group. With this in mind, the results in the present study where the health gap in terms of higher dft/DFT, more extractions, and fewer dental examinations increases during placement it is troublesome. While causality cannot be established, the results show that children in OHC have poorer dental health than their peers. Once placed, they often lack access to dental examinations as required by law. This increases the risk that poor dental health at the time of placement may go unnoticed, and even continues to deteriorate as many children in OHC do not receive the necessary care and support. Apparently, society has not been able to buffer or compensate for the health inequalities. Rather, children in OHC have been let down.

### Strengths and weaknesses

This study was based on a well-defined cohort, the Swedish Out of Home Care Children cohort (SweOHC), and included all children placed in OHC during a specified study period. Further, to enable valid comparisons with the background population. five controls, matched for age, sex and county region, were identified for each child in the OHC group. This design and the high number of individuals included are important strengths of the present study. Another strength was the similarity in dental health between controls in this study and the official statistics on dental health published annually by the Swedish National Board of Health and Welfare [28], indicating that the controls reflect the background population well.

There are inherent limitations in a study such as this. As a registry-based study, information about risk factors for dental caries, such as diet or oral hygiene, are not available, nor do we have any information about the children’s perspectives. Confounding factors are almost always present in observational studies such as this one. To address this, we included sociodemographic variables and comorbidities that may be associated with both OHC and oral health outcomes in our adjusted analyses. The weaker associations observed between OHC and dental caries after adjustment suggest that a substantial portion of the initial association was explained by these confounding factors. However, a potential limitation that complicates interpretation is the risk of residual confounding, which may persist even after statistical adjustments. Furthermore, detailed information as to why the children were placed in OHC was unavailable, as the registries do not include that data. Moreover, as the main focus in this study was dental health, it is a further shortcoming that around 11% of the study population could not be found in the SKaPa-registry. There are two different explanations: the child has been treated by a dental healthcare provider who is not affiliated with SKaPa (the number of private dental practitioners who are not in SKaPa is still quite high), or the child has not had a dental appointment at all during the period, 2010–2020. However, the proportions of missing individuals were similar in the control and the OHC group and as the number of individuals included in the analysis was generally high it is fair to assume that the results are reliable. Further, this is in line with a Swedish report showing that 13% of adolescents miss their dental appointments [29]. The study period also covered the year 2020 when the COVID-19 pandemic impacted on children’s access to dental care [30].

## Conclusion

This study reveals that children placed in out-of-home care in Sweden have poorer dental health than others and that there are major shortcomings in ensuring that these children receive the dental care to which they are entitled. The dental health needs of children entering or already placed in OHC should be met more effectively than they are today. There is a difference in dental health assessments before and after the year 2017, with higher frequencies of dental health assessments after the legislative amendment in 2017. There is a need for reappraisal of guidelines, legislation, and organizational models for providing dental care to children and adolescents in out-of-home care.

## Data Availability

Researchers have the possibility of applying for the datasets used and/or analyzed from the corresponding author on reasonable request. The data was retrieved from The National Board of Health and Welfare, Statistics Sweden and the SKaPa registry after ethical approval and application to the registries.

## Abbreviations

CRC: United Nations Convention on the Rights of the Child
DEFT: Decayed, extracted, filled teeth (primary teeth)
DFT: Decayed, filled teeth (primary teeth)
DFT: Decayed, Filled Teeth (permanent teeth)
DMFT: Decayed, Missing, Filled Teeth (permanent teeth)
LISA: Longitudinal Integration Database for Health Insurance and Labour Market Studies
OHC: Out-of-home care
PIN: Personal identity number
SKaPa: The Swedish Quality Registry for Caries and Periodontal Diseases
SweOHC: Swedish Out-of-Home Care Children cohort
